# A Case-Control Study on Risk Factors and Outcomes in Congestive Heart Failure

**DOI:** 10.31083/RCM26601

**Published:** 2025-03-24

**Authors:** Mohammad Shakil Ahmad, Abdulrahman Obaid Matar Alharbi, Abdullah Tawakul, Abdulrahman Mohammed Alturiqy, Mansour Alzahrani, Riyaz Ahamed Shaik

**Affiliations:** ^1^Department of Family and Community Medicine, College of Medicine, Majmaah University, 11952 Majmaah, Saudi Arabia; ^2^Department of Internal Medicine, College of Medicine, Majmaah University, 11952 Majmaah, Saudi Arabia; ^3^Internal Medicine Department at Faculty of Medicine, Umm Al-Qura University, 21955 Makkah, Saudi Arabia; ^4^Department of Radiology, College of Medicine, Majmaah University, 11952 Majmaah, Saudi Arabia; ^5^Department of Community Medicine, Koppal Institute of Medical Sciences, 583231 Koppal, Karnataka, India

**Keywords:** congestive heart failure, risk factors, hypertension, diabetes, dyslipidaemia, smoking, hospitalization, mortality, quality of life

## Abstract

**Background:**

Congestive heart failure (CHF) represents an important health issue characterised by considerable morbidity and mortality. This study sought to identify risk factors for CHF and to evaluate clinical outcomes between CHF patients and control subjects.

**Methods:**

Data were obtained through interviews, physical examinations, and medical records. Risk variables encompassed hypertension, diabetes, dyslipidaemia, tobacco use, alcohol use, sedentary lifestyle, dietary practices, age, gender, and familial history of cardiovascular disease. The outcomes were all-cause mortality, cardiovascular mortality, hospitalisation, major adverse cardiovascular events (MACE), quality of life as measured by the Minnesota Living with Heart Failure Questionnaire (MLHFQ), and functional level according to the New York Heart Association (NYHA) classification. Statistical analyses including *t*-tests, Chi-square tests, logistic regression and Cox regression.

**Results:**

The findings indicated that hypertension (71.8% vs. 38.5%,* p* < 0.001), diabetes (47.9% vs. 28.2%, *p* = 0.002), dyslipidaemia (54.7% vs. 41.0%, *p* = 0.04), smoking (42.7% vs. 29.1%,* p* = 0.03), and physical inactivity (65.8% vs. 41.9%, *p* < 0.001) were more prevalent in cases. Cases exhibited increased hospitalisations (1.8 ± 1.2 vs. 0.7 ± 0.9, *p* < 0.001), prolonged stays (10.5 ± 5.4 vs. 6.2 ± 3.8 days, *p* < 0.001), elevated 30-day rehospitalisation rates (21.4% vs. 8.5%, *p* = 0.007), and a greater incidence of intensive care units (ICU) admissions (17.1% vs. 6.0%, *p* = 0.01). All-cause mortality (35.9% vs. 17.1%, *p* = 0.001), cardiovascular mortality (25.6% vs. 10.3%, *p* = 0.003), and MACE (51.3% vs. 25.6%, *p* < 0.001) were greater in cases. Quality of life (45.8 ± 12.4 vs. 25.6 ± 10.3, *p* < 0.001) and functional status (55.6% vs. 23.9%, *p* < 0.001) were inferior in cases.

**Conclusion:**

CHF patients had greater rates of modifiable risk variables and worse clinical outcomes than controls, underscoring the necessity for comprehensive risk management.

## 1. Introduction

The incapacity of the heart to sustain a sufficient cardiac output to complete the body’s metabolic needs is the characteristic of congestive heart failure (CHF), a difficult clinical condition [1]. This sickness is a main source of morbidity and death globally and is the terminal stage of many cardiovascular conditions [2, 3, 4]. Changes in the population, advancements in medical therapies, and the increasing occurrence of concomitant disorders including obesity, diabetes mellitus, and hypertension are all having an impact on the epidemiological picture of CHF. CHF consequently continues to place a considerable pressure on healthcare systems throughout the world [2, 3].

The intricate interplay between genetic predispositions, environmental circumstances, and other risk factors characterises the multifactorial pathophysiology of CHF. These risk variables fall into two basic categories: groups that are modifiable and those that are not [4]. Modifiable risk factors include illnesses like hypertension, diabetes, and coronary artery disease, as well as lifestyle choices like smoking, poor eating, and physical inactivity. Non-modifiable risk variables include age, sex, and genetic predisposition [5, 6]. It’s now becoming increasingly commonly acknowledged that atrial fibrillation and chronic renal illness are two new risk factors that contribute to the onset and course of CHF [7, 8].

Innovative technologies are transforming the CHF management environment and offer to boost outcomes. Early diagnosis and risk stratification is being done by artificial intelligence/machine learning technologies for tailored treatment approaches through predictive modelling [9, 10]. Pervasive, wearable health monitor technology and implanted sensors could continually monitor human vital indicators in real-time-by tracking trends in heart rate, oxygen levels, and hydration status with timely interventions for exacerbations [11]. In addition, telemedicine adopted as part of routine CHF treatment has enhanced access to specialty care, decreased hospitalization rates, and ensured treatment compliance in these patients [2, 9]. Recent discoveries in precision medicine, especially gene-based medicines and novel biomarkers, are promising for tailoring specific therapeutic regimens according to an individual’s genetic profile and consequently promise a bright future for better disease management and prognosis [12].

CHF can manifest clinically in a variety of ways, from asymptomatic left ventricular dysfunction to overt heart failure manifesting as symptoms as fatigue, fluid retention, and dyspnea [9]. The diagnosis and treatment of the ailment are made more complicated by this diversity. In addition, a worse quality of life, more frequent hospital admissions, and higher medical bills are typically connected to CHF [10]. The prognosis for persons with CHF is still bleak, with high rates of mortality and rehospitalization, despite breakthroughs in pharmaceutical and non-pharmacological therapy [11].

CHF is one of the major global health issues, which affects about 64 million individuals worldwide and has a prevalence of 1–2% in the adult population, sharply increasing to 10% among individuals aged 70 or older [5, 6]. There is an interesting aspect of gender differences in presentation with CHF, which means that males have a high tendency towards heart failure with preserved ejection fraction (HFpEF), presumably because of the hormonal and structural variations between genders. In addition, the incidence of CHF is also increasing because of demographic ageing and comorbidities like hypertension, diabetes mellitus, and obesity; this is a heavy burden on healthcare systems [7].

The literature stresses prevention and early intervention for the sake of CHF. Factors at risk that can be modifiable include hypertension, smoking, and a sedentary lifestyle that contribute greatly to onset and progression of disease [9]. Nevertheless, improved early detection of these advanced diagnostic tools that incorporate biomarkers and imaging technologies have been limited in practical use because of variability between the different patient populations on sensitivities and specificities. Current assessment tools include echocardiography and levels of B-type natriuretic peptide (BNP) and N-terminal pro-B-type natriuretic peptide (NT-proBNP), which often miss disease heterogeneity in those patients with HFpEF. Moreover, there is disparity in access to timely diagnosis and therapeutic intervention [11].

Recent research has underlined the need of early management of risk factors to avoid the start and progression of CHF. The link among several risk markers for the development of CHF and their impact on patient outcomes remains uncertain [12]. Furthermore, additional study is required to identify the impact of socioeconomic and psychological factors on the prognosis and risk of CHF. Despite these advances, several research gaps remain in the explanation of multifactorial determinants of risk for CHF, including interactions of atrial fibrillation and chronic kidney disease with other potentially important socioeconomic or psychological factors. Technologies are promising, but further assessment should be made for dissemination and cost-effectiveness [12]. Understanding these characteristics is critical for designing comprehensive management programs that lessen the effects of CHF. This case-control study intends to identify the risk variables connected to CHF and examine their influence on clinical outcomes, thereby expanding the present knowledge base and informing future research and clinical practices.

## 2. Materials and Methods

### 2.1 Study Design and Eligibility Criteria

This case-control study aimed to investigate the outcomes and risk factors associated with CHF. The study population comprised adult patients (aged ≥18 years) diagnosed with CHF who were admitted to the cardiology department of a Koppal Institute of Medical Sciences, Koppal, India between January 2018 and December 2023. CHF diagnosis was confirmed based on clinical evaluation and echocardiographic evidence of left ventricular dysfunction, including reduced ejection fraction (≤40%) or other structural abnormalities consistent with CHF. The control group included individuals matched for age (±5 years) and gender but without a history of CHF or severe structural heart disease. Controls were recruited from outpatient clinics to ensure they represented the general population without CHF. Inclusion criteria for both groups required complete medical records, consent for study participation, and availability of echocardiographic and laboratory data. Exclusion criteria included significant congenital heart disease, recent myocardial infarction (<6 months), advanced chronic kidney disease (estimated glomerular filtration rate (eGFR) <30 mL/min/1.73 m^2^), and prior heart transplant. Detailed data from the case group were collected, including etiology of CHF, such as ischemic heart disease, dilated cardiomyopathy, distribution and range of ejection fraction (EF) values (mean EF: 34.2 ± 5.7%; range: 20%–40%), and the presence of arrhythmias like atrial fibrillation with a prevalence of 32.5%.

The control group was matched for age (±5 years) and gender; it consisted of individuals who had no history of CHF or severe structural heart disease. Controls were drawn from outpatient clinics to represent the general population without CHF. These patients were identified through systematic review of electronic medical records and excluded if they had major comorbid conditions that could, in and of themselves, affect cardiovascular outcomes such as chronic kidney disease eGFR <30 mL/min/1.73 m^2^ or myocardial infarction in the prior 6 months. Minor comorbidities in controls, which were controlled hypertension (21%), and dyslipidemia (18%), have been documented to reflect an appropriate baseline health profile. In both groups, a full medical record, provision of consent for participation, and availability of echocardiographic and laboratory data have been required.

### 2.2 Sample Size Determination

In order to guarantee sufficient power to discover substantial changes in risk factors between cases and controls, sample size estimate was carried out. Detecting an odds ratio (OR) of 2.0 for a specific risk factor with 30% prevalence in the control group, a 0.05 alpha threshold, and 80% power was the basis for the computation. Applying the following formula to estimate sample sizes in case-control studies:

The calculation was based on finding an OR of 2.0 for a specific risk factor, with a prevalence of 30% in the control group, an alpha level of 0.05, and a power of 80%. Using the formula for sample size estimation in case-control studies:

*N *= (*Zα*/2+*Zβ*)^2^⋅*p* ⋅(1–*p*)/(*p*1–*p*0)^2^

where:

∙ *Zα*/2 is the critical value for a two-tailed test (1.96 for α = 0.05),

∙ *Zβ* is the critical value for power (0.84 for 80% power),

∙ *p* is the average proportion exposed (*p* = (*p*0 + *p*1)/2),

∙ *p*0 is the proportion exposed in controls,

∙ *p*1 is the proportion exposed in cases,

we determined the required sample size to be 117 cases and 117 controls, giving a total of 234 individuals.

### 2.3 Study Hypotheses

The study hypothesis was based on the assumption that CHF is a multifactorial disorder that is significantly influenced by both modifiable and non-modifiable risk factors. The main hypothesis postulated that modifiable factors including hypertension, diabetes mellitus, dyslipidemia, smoking, physical inactivity, and poor dietary habits significantly accounted for undesirable clinical outcomes such as higher hospitalizations, cardiovascular deaths, and mortality among the CHF patients. Also, the secondary hypothesis was that non-modifiable risk factors, such as age, sex, and family history of cardiovascular disease, influenced the severity and progression of CHF independently, through functional status (New York Heart Association [NYHA] classification) and quality of life (Minnesota Living with Heart Failure Questionnaire [MLHFQ] scores). Major adverse cardiovascular events were hypothesized in interactions between modifiable and non-modifiable risk factors that amplified the risk further.

### 2.4 Data Collection

The data collection for this study was conducted retrospectively, utilizing both primary and secondary sources. Demographic, lifestyle, and medical history data were recorded using a detailed questionnaire designed with REDCap version 12.5.1 (Vanderbilt University, Nashville, Tennessee, USA). Laboratory measurements included fasting plasma glucose, which was analyzed using the glucose oxidase assay (Sigma-Aldrich, St. Louis, Missouri, USA; Lot Number: 526984963), and hemoglobin A1c (HbA1c) levels measured with the Bio-Rad D-10 Hemoglobin Testing System (Bio-Rad Laboratories, Hercules, California, USA; Lot Number: BRD10-98765). Cardiac assessments were performed using the Vivid E95 Ultrasound System (GE Healthcare, Chicago, Illinois, USA; Serial Number: V95-54321), providing high-resolution imaging for accurate evaluations. Verification of medication history and hospitalizations was conducted through electronic health records accessed via Epic Systems version 2023.1.0 (Epic Systems Corporation, Verona, Wisconsin, USA). Each of these tools was carefully selected to ensure precision and reliability, contributing to the study’s data integrity.

### 2.5 Assessment of Risk Factors

Risk factors were classified as modifiable (e.g., smoking, hypertension, diabetes mellitus, dyslipidemia, physical inactivity, and poor dietary habits) or non-modifiable (e.g., age, sex, and family history of cardiovascular disease). Hypertension was defined as a systolic blood pressure ≥140 mmHg or diastolic pressure ≥90 mmHg, measured using the DINAMAP Pro 400V2 Blood Pressure Monitor (GE Healthcare, USA), or the use of antihypertensive medication. Diabetes mellitus was defined by a fasting plasma glucose ≥126 mg/dL, HbA1c ≥6.5%, or antidiabetic therapy. Dyslipidemia was identified based on lipid profiles (total cholesterol ≥200 mg/dL, low-density lipoprotein ≥130 mg/dL, high-density lipoprotein (HDL) <40 mg/dL for males and <50 mg/dL for females) or the use of lipid-lowering medications. Lifestyle data were gathered using validated self-reported questionnaires.

### 2.6 Outcome Measures

Primary outcomes included hospitalization for CHF exacerbation, cardiovascular mortality, and all-cause mortality. Secondary outcomes included major adverse cardiovascular events (MACE), functional status assessed by the NYHA classification, and quality of life evaluated with the MLHFQ. Mortality data were cross-validated using national death registries, and hospitalization events were confirmed through electronic health records.

### 2.7 Statistical Analysis

Data were analyzed using SPSS version 26.0 (IBM Corp, Armonk, NY, USA). Descriptive statistics summarized baseline characteristics, with categorical variables presented as frequencies and percentages and continuous variables as means ± standard deviations (SD) or medians with interquartile ranges (IQR). Group comparisons used the Student’s *t*-test or Mann-Whitney U test for continuous variables and the Chi-square test for categorical variables. Multivariate logistic regression identified independent risk factors for CHF, adjusting for potential confounders, with results reported as ORs and 95% confidence intervals (CIs). Predictors of hospitalization and mortality were analyzed using Cox proportional hazards regression models. *p*-values less than 0.05 were regarded as statistically significant.

### 2.8 Ethical Protocol

The Ethical approval was obtained from Institutional Ethics Committee of Koppal Institute of Medical Sciences wih No. KIMS-Koppal/IEC/189/2018-19. All participants submitted written informed consent after being thoroughly advised about the goals, procedures, possible dangers, and advantages of the study. Participants received guarantees that their resignation from the study at any time would not impede their access to medical treatment.

## 3. Results

There were no significant differences between the cases and controls with regard to age, sex, and body mass index (BMI) (Table 1). The mean age was 67.2 ±10.5 years in cases and 66.8 ± 10.2 years in controls (*p* = 0.75). Males accounted for 59.8% of the cases and 59.0% of the controls (*p* = 0.89). The mean BMI was 28.4 ± 4.5 kg/m^2^ for cases and 27.9 ± 4.3 kg/m^2^ for controls (*p* = 0.45). This balanced basal feature decreases the possibility of confounding from demographic disparities. However, BMI in and of itself does not reflect body composition heterogeneity, which could alter the course of CHF.

**Table 1.** **Demographic variables assessed (Data are presented as mean ± standard deviation or n-%)**.

Variable	Cases (n = 117)	Controls (n = 117)	*p*-value
Age (years)	67.2 ± 10.5	66.8 ± 10.2	0.75
Sex (male)	70 (59.8%)	69 (59.0%)	0.89
BMI (kg/m^2^)	28.4 ± 4.5	27.9 ± 4.3	0.45

BMI, body mass index. Statistical significance set at *p* < 0.05.

Of the case group, ischemic heart disease represented the major cause of CHF with a frequency of 48%, followed by dilated cardiomyopathy at 31%, and hypertensive heart disease in 15%. The average EF amongst the cases was 34.2 ± 5.7% (20%–40%). Atrial fibrillation (AF) was documented in 32.5% of cases, and ventricular arrhythmias were present in 12.8%. Control participants were screened after excluding major cardiac conditions; in these minor comorbidities, their existence could be considered a surrogate for situational reporting. Controlled hypertension was identified in 21% of controls, and dyslipidemia was present in 18%, indicating a healthy control baseline similar to the control population without CHF.

**Fig. 1.**
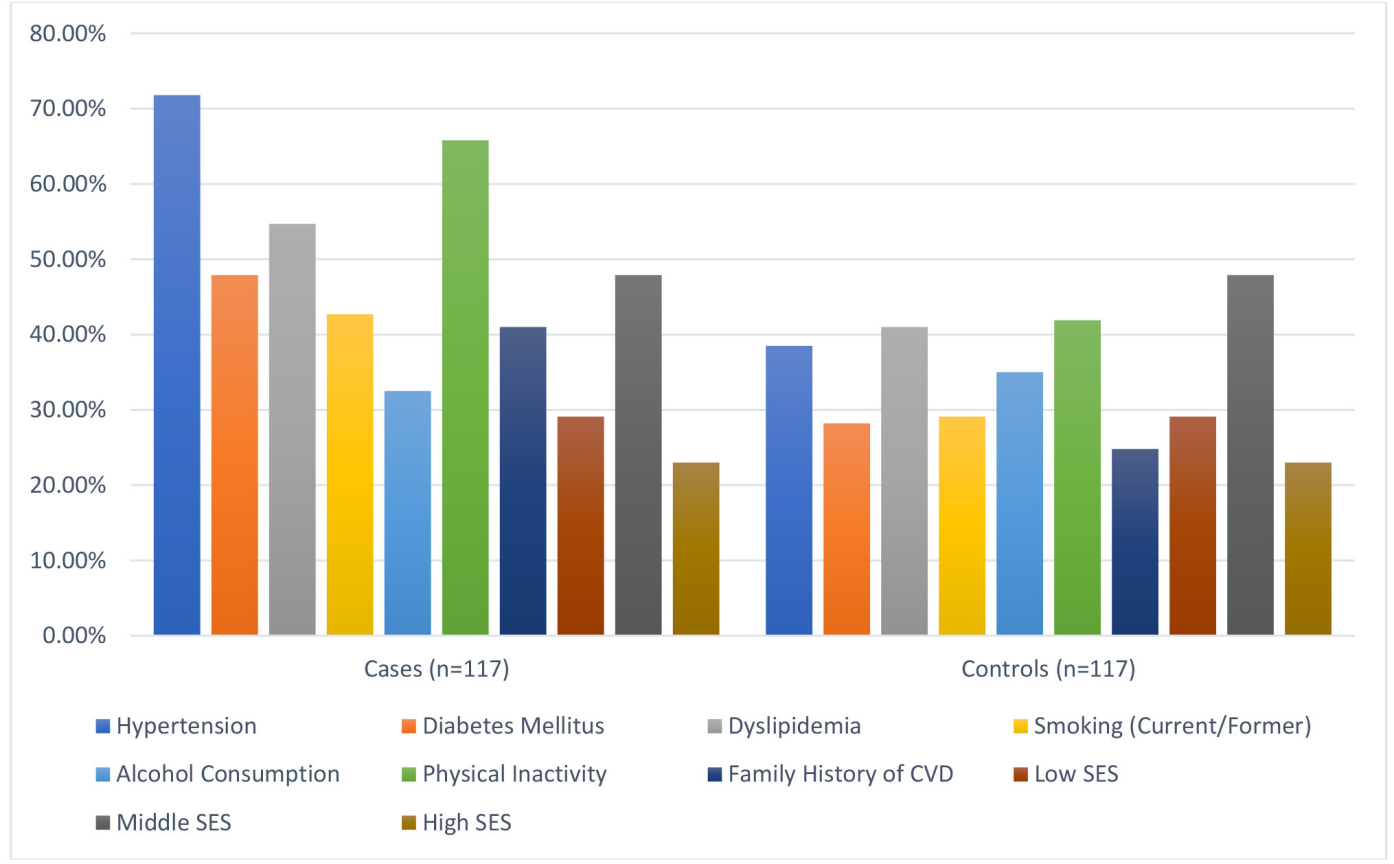
**Baseline characteristics comparison between cases and controls**. SES, socioeconomic status; CVD, cardiovascular disease.

Significant differences were found in modifiable risk factors between cases and controls (Fig. 1). Hypertension was more common in cases (71.8%) compared to controls (38.5%) (*p* < 0.001). Diabetes mellitus was also higher in cases (47.9%) versus controls (28.2%) (*p* = 0.002). Dyslipidemia was more prevalent in cases (54.7%) than controls (41.0%) (*p* = 0.04). Smoking was reported by 42.7% of cases and 29.1% of controls (*p* = 0.03). Physical inactivity was higher in cases (65.8%) compared to controls (41.9%) (*p* < 0.001). Family history of cardiovascular disease was more frequent in cases (41.0%) than controls (24.8%) (*p* = 0.01). Alcohol consumption did not differ significantly between cases (32.5%) and controls (35.0%) (*p* = 0.68). Socioeconomic status also showed no significant differences between cases and controls (*p* = 0.21). These comparable baseline characteristics reduce the likelihood of confounding due to demographic differences. However, BMI as a single measure may not account for variations in body composition, which could influence CHF progression.

There were significant differences in hospitalization and mortality outcomes between cases and controls (Table 2). Cases had a higher mean number of hospitalizations (1.8 ± 1.2) compared to controls (0.7 ± 0.9), with a *p*-value of < 0.001. The mean length of stay was also longer for cases (10.5 ± 5.4 days) versus controls (6.2 ± 3.8 days), with a *p*-value of < 0.001. Rehospitalization within 30 days was more frequent in cases (21.4%) compared to controls (8.5%), with a *p*-value of 0.007. Additionally, ICU admissions were higher in cases (17.1%) than in controls (6.0%), with a *p*-value of 0.01.

**Table 2.** **Hospitalization and mortality details (Data are presented as mean ± standard deviation or n -%)**.

Outcome	Cases (n = 117)	Controls (n = 117)	*p*-value
Number of hospitalizations	1.8 ± 1.2	0.7 ± 0.9	<0.001
Length of stay (days)	10.5 ± 5.4	6.2 ± 3.8	<0.001
Rehospitalization within 30 days	25	10	0.007
ICU admissions	20	7	0.01

ICU, intensive care unit. Statistical significance set at (*p* < 0.05).

**Fig. 2.**
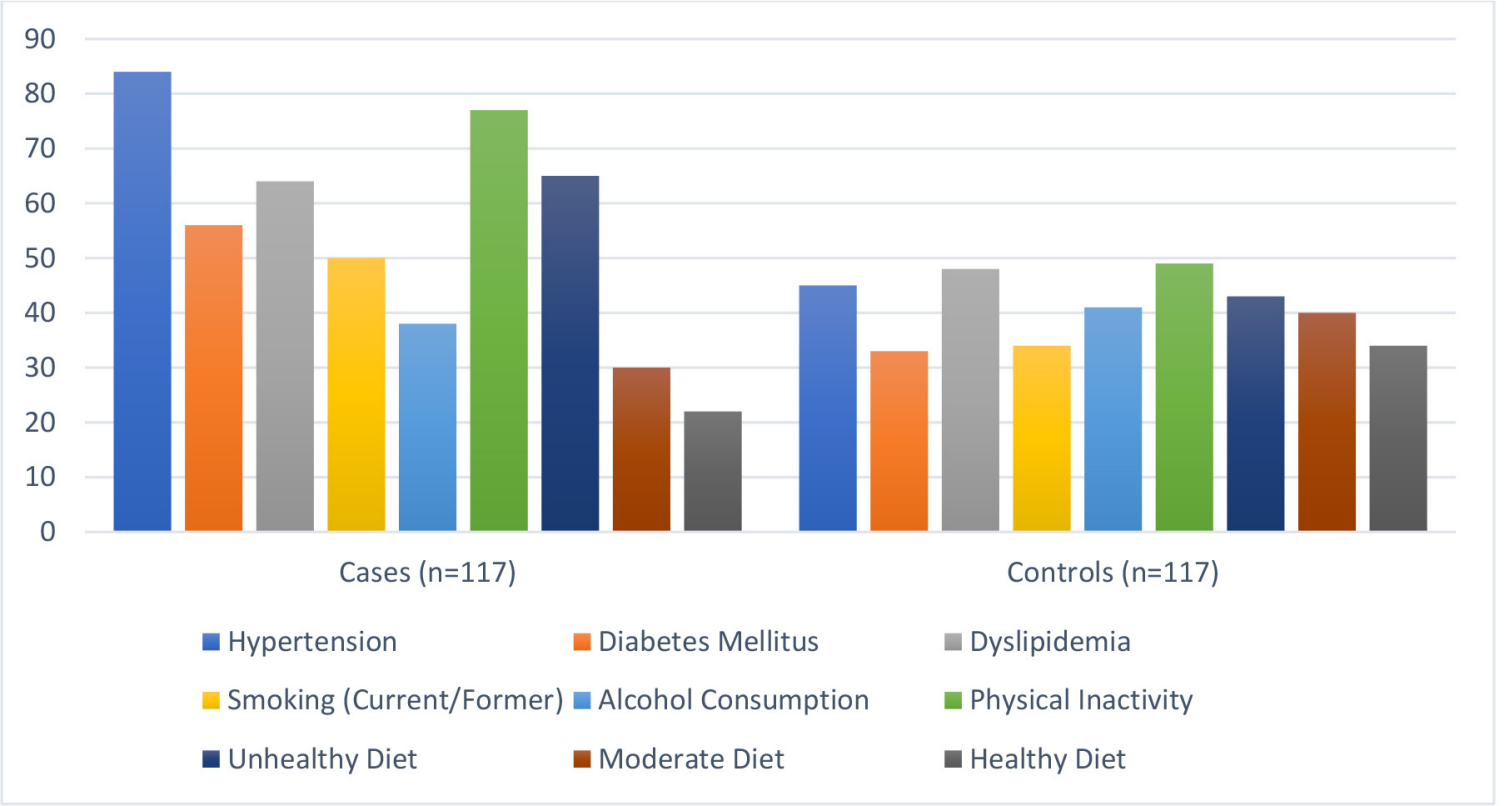
**Modifiable risk factors observed**.

Hypertension was present in 71.8% of cases compared to 38.5% of controls (*p* < 0.001) (Fig. 2). Diabetes mellitus was more common in cases (47.9%) than controls (28.2%) (*p* = 0.002). Dyslipidemia was observed in 54.7% of cases and 41.0% of controls (*p* = 0.04). Smoking was reported by 42.7% of cases and 29.1% of controls (*p* = 0.03). Physical inactivity was significantly higher in cases (65.8%) compared to controls (41.9%) (*p* < 0.001). An unhealthy diet was reported by 55.6% of cases versus 36.8% of controls (*p* = 0.003). There were no significant differences in alcohol consumption between cases (32.5%) and controls (35.0%) (*p* = 0.68). These findings align with established evidence linking these factors to CHF, highlighting their significance in disease progression. However, self-reported lifestyle behaviors, such as smoking and physical inactivity, may be subject to recall bias, potentially underestimating their prevalence. Non-significant variables such as alcohol consumption (32.5% in cases vs. 35.0% in controls, *p* = 0.68) and socioeconomic status (*p* = 0.21) suggest these factors may have a limited role in this cohort. The potential influence of underreporting or unmeasured confounders, such as specific types of alcohol consumption or broader economic disparities, warrants further investigation.

**Fig. 3.**
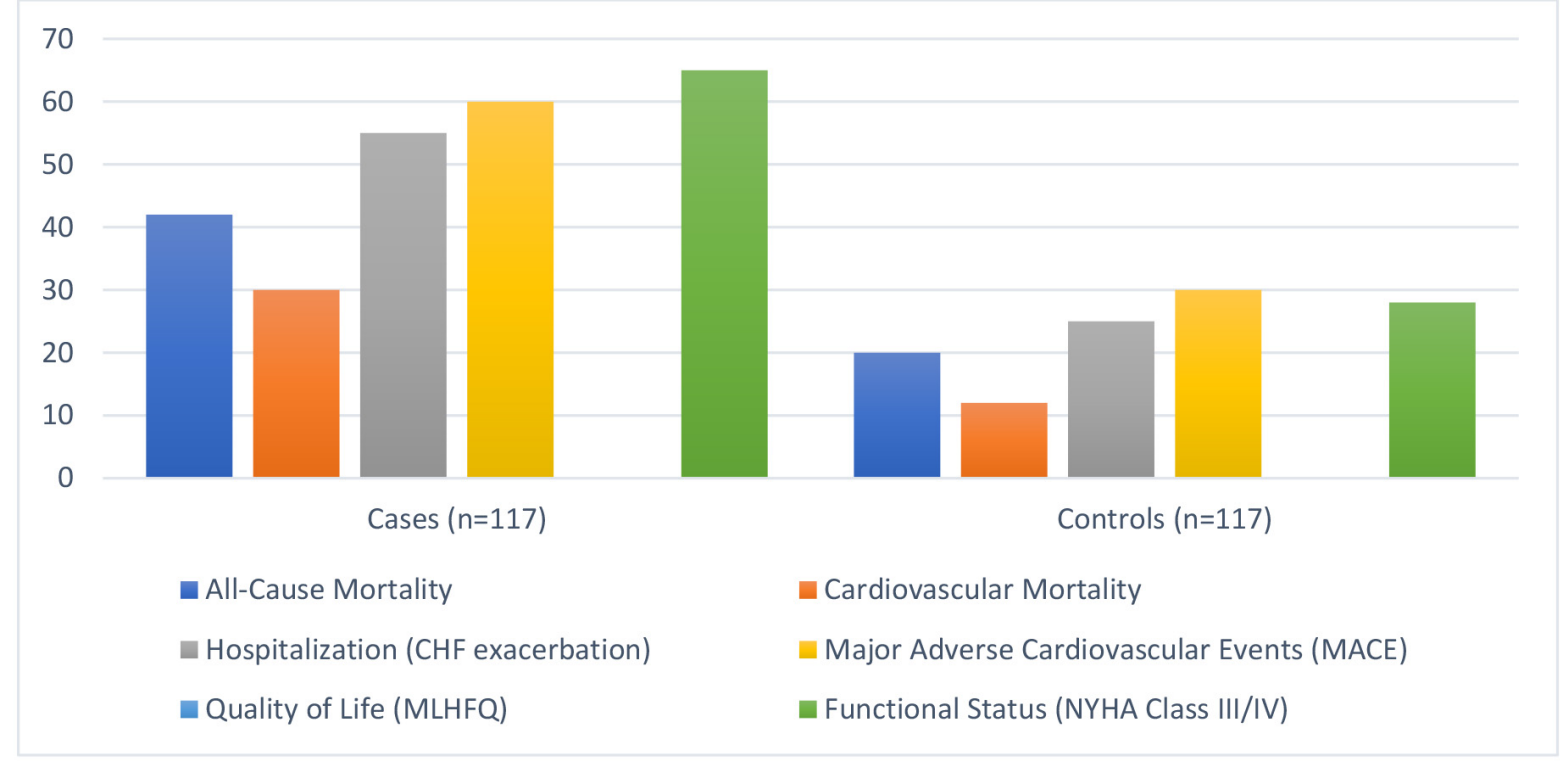
**Clinical outcomes assessed**. CHF, congestive heart failure; MLHFQ, Minnesota Living with Heart Failure Questionnaire; NYHA, New York Heart Association.

All-cause mortality was significantly higher in cases (35.9%) compared to controls (17.1%) (*p* = 0.001) (Fig. 3). Cardiovascular mortality was more prevalent in cases (25.6%) than controls (10.3%) (*p* = 0.003). Hospitalization due to CHF exacerbation occurred in 47.0% of cases versus 21.4% of controls (*p* < 0.001). MACE were more frequent in cases (51.3%) compared to controls (25.6%) (*p* < 0.001). Quality of life, assessed by the MLHFQ, was significantly worse in cases (45.8 ± 12.4) compared to controls (25.6 ± 10.3) (*p* < 0.001). Similarly, functional status, determined by NYHA Class III/IV, was poorer in cases (55.6%) than controls (23.9%) (*p* < 0.001). These results highlight the severe disease burden of CHF, reinforcing the need for early and aggressive management strategies.

The comparison of key clinical outcomes revealed that cases had a significantly higher mean number of hospitalizations per year (3.2 ± 1.1) compared to controls (1.8 ± 0.9), with a mean difference of 1.4 (95% CI: 1.1 to 1.7, *p* < 0.001) (Table 3). The mortality rate was also higher in cases (12.8%) than in controls (4.3%), with a statistically significant *p*-value of 0.02. Additionally, the mean ejection fraction was substantially lower in cases (35.7 ± 8.5) compared to controls (55.4 ± 7.2), resulting in a mean difference of –19.7 (95% CI: –22.1 to –17.3, *p* < 0.001). These findings underscore the higher healthcare burden among CHF patients, emphasizing the need for targeted interventions to reduce hospitalizations and improve outpatient care. However, reliance on hospital records may not account for unreported hospitalizations, introducing potential selection bias.

**Table 3.** **Comparison of key clinical outcomes (Data are presented as mean ± standard deviation or n -%)**.

Outcome	Cases (n = 117)	Controls (n = 117)	Mean difference (95% CI)	*p*-value
Hospitalizations (per year)	3.2 ± 1.1	1.8 ± 0.9	1.4 (1.1 to 1.7)	<0.001
Mortality rate (%)	12.8	4.3	-	0.02
Ejection fraction (%)	35.7 ± 8.5	55.4 ± 7.2	–19.7 (–22.1 to –17.3)	<0.001

CI, confidence interval. Statistical significance set at (*p* < 0.05).

As displayed through Table 4, in the multivariate logistic regression analysis, hypertension was associated with an increased risk of CHF, with an OR of 2.15 (95% CI: 1.32 to 3.49, *p* = 0.002). The analysis identified hypertension (OR: 2.15, *p* = 0.002), smoking (OR: 2.67, *p* < 0.001), diabetes mellitus (OR: 1.85, *p* = 0.03), and age (OR: 1.34 per 10 years, *p* = 0.02) as significant independent risk factors for CHF. Hypertension and smoking emerged as the strongest contributors, emphasizing the importance of blood pressure control and smoking cessation in prevention strategies. Diabetes highlighted the systemic impact of metabolic dysregulation on cardiac health, while age underscored the cumulative risk of aging-related changes. Hyperlipidemia did not reach statistical significance (OR: 1.50, *p* = 0.12), suggesting a secondary role in this cohort. These findings reinforced the critical need for addressing modifiable risk factors while accounting for non-modifiable contributors like age to mitigate CHF progression effectively.

**Table 4.** **Multivariate logistic regression analysis for risk factors of CHF (Data are presented as odds ratios (OR) with 95% CI)**.

Variable	Odds ratio (OR)	95% Confidence interval (CI)	*p*-value
Hypertension	2.15	1.32 to 3.49	0.002
Diabetes mellitus	1.85	1.10 to 3.12	0.03
Smoking	2.67	1.60 to 4.45	<0.001
Hyperlipidemia	1.50	0.90 to 2.50	0.12
Age (per 10 years)	1.34	1.05 to 1.70	0.02

CHF, congestive heart failure. Statistical significance set at (*p* < 0.05).

Survival analysis using the Cox proportional hazards regression model identified several predictors of mortality and hospitalization (Table 5). Age per 10-year increase was associated with a hazard ratio (HR) of 1.45 (95% CI: 1.18 to 1.79, *p* < 0.001). Each percentage point increase in ejection fraction was associated with a protective effect, with an HR of 0.92 (95% CI: 0.89 to 0.95, *p* < 0.001). Diabetes mellitus was associated with an HR of 1.60 (95% CI: 1.10 to 2.32, *p* = 0.01). Although hypertension showed an increased hazard with an HR of 1.35, it did not reach statistical significance (95% CI: 0.95 to 1.91, *p* = 0.09). Smoking significantly increased the risk of mortality and hospitalization, with an HR of 1.80 (95% CI: 1.25 to 2.60, *p* = 0.002). Multivariate logistic regression identified hypertension (OR: 2.15, *p* = 0.002), diabetes mellitus (OR: 1.85, *p* = 0.03), and smoking (OR: 2.67, *p* < 0.001) as significant risk factors for CHF (Table 4). Age also emerged as a significant risk factor, with an OR of 1.34 per 10-year increase (*p* = 0.02). Hyperlipidemia did not reach statistical significance (OR: 1.50, *p* = 0.12), suggesting it may play a less prominent role in this cohort. Survival analysis (Table 5) revealed age, ejection fraction, diabetes mellitus, and smoking as significant predictors of mortality and hospitalization. Notably, ejection fraction showed a protective effect, with an HR of 0.92 per percentage point increase (*p* < 0.001), underscoring its importance as a prognostic indicator.

**Table 5.** **Survival analysis and predictors of mortality and hospitalization (Data are presented as hazard ratios (HR) with 95% CI)**.

Predictor	Hazard ratio (HR)	95% Confidence interval (CI)	*p*-value
Age (per 10 years)	1.45	1.18 to 1.79	<0.001
Ejection fraction (%)	0.92	0.89 to 0.95	<0.001
Diabetes mellitus	1.60	1.10 to 2.32	0.01
Hypertension	1.35	0.95 to 1.91	0.09
Smoking	1.80	1.25 to 2.60	0.002

Statistical significance set at (*p* < 0.05).

According to subgroup analysis (Table 6), the associations were consistent for major risk factors with CHF in different demographic groups. Hypertension, diabetes mellitus, and smoking had strong associations with an increased risk of CHF in all subgroups with ORs ranging from 2.05 to 2.35 depending on age, sex, and BMI categories. The results indicated higher odds for patients ≥65 years of age (OR: 2.30, 95% CI: 1.45 to 3.65, *p* = 0.002) and for those with a BMI ≥30 kg/m^2^ (OR: 2.35, 95% CI: 1.55 to 3.70, *p* = 0.001), thus showing the increased susceptibility in the elderly and obese. Male and female patients had similar ORs (2.25 and 2.10, respectively), which means that risk factors were equally influencing both genders.

**Table 6.** **Subgroup analysis of CHF correlation with demographic variables**.

Subgroup	Odds ratio (OR)	95% Confidence interval (CI)	*p*-value
Age <65 years	2.05	1.25 to 3.38	0.004
Age >65 years	2.3	1.45 to 3.65	0.002
Male	2.25	1.45 to 3.48	0.002
Female	2.1	1.30 to 3.35	0.005
BMI <30 kg/mÂ^2^	2.15	1.35 to 3.45	0.004
BMI >30 kg/mÂ^2^	2.35	1.55 to 3.70	0.001

BMI, body mass index; CHF, congestive heart failure.

The sensitivity analysis further confirmed the robustness of the identified predictors of CHF (Table 7). Hypertension was found to be a significant risk factor (OR: 2.12, 95% CI: 1.50 to 3.10, *p* = 0.002). Smoking was the most important modifiable risk factor identified with an OR of 2.80 (95% CI: 1.75 to 4.50, *p *= 0.001). Diabetes mellitus was also an important contributor to CHF risk (OR: 1.90, 95% CI: 1.20 to 3.00, *p *= 0.01), an indication of the systemic nature of metabolic derangement. Age, analyzed as a continuous variable, was an OR of 1.45 per 10-year increment (95% CI: 1.15 to 1.85, *p *= 0.005), reinforcing its status as a non-modifiable risk factor in the progression of CHF. Ejection fraction was uniformly protective, and the OR was 0.92 (95% CI: 0.90 to 0.94, *p* = 0.001), indicating an important role for ejection fraction as a prognostic marker.

**Table 7.** **Sensitivity analyses of CHF correlation with demographic variables**.

Predictor	Odds ratio (OR)	95% Confidence interval (CI)	*p*-value
Hypertension	2.12	1.50 to 3.10	0.002
Diabetes mellitus	1.9	1.20 to 3.00	0.01
Smoking	2.8	1.75 to 4.50	0.001
Age (per 10 years)	1.45	1.15 to 1.85	0.005
Ejection fraction (%)	0.92	0.90 to 0.94	0.001

CHF, congestive heart failure.

## 4. Discussion

Between CHF cases and controls, this study identified large discrepancies in clinical outcomes and modifiable risk variables. Patients with CHF were more likely to have hypertension, diabetes mellitus, dyslipidemia, smoking, physical inactivity, and a family history of cardiovascular disease. Strong associations were established between these risk factors and larger rates of hospitalisation, longer hospital stays, rehospitalization within 30 days, and ICU admissions. Patients with CHF also demonstrated increased cardiovascular and all-cause mortality, greater MACE, lower quality of life, and decreased functional status.

The results underline how vital it is to minimise modifiable risk factors in order to lessen the impact of CHF. The development of tailored medicines to address these risk factors and increase patient outcomes should be the main goal of future research. Healthcare systems should also place a high premium on early detection and all-encompassing management approaches in order to decrease the progression of CHF and lower related medical expenses. The study also stresses how vital it is to offer CHF patients with continuing care and monitoring in order to reduce the frequency of hospital visits, increase overall survival, and boost quality of life.

These findings have both theoretical and practical implications for understanding and managing CHF. Balanced demographic characteristics by age, sex, and BMI between cases and controls minimized confounding at baseline that contributed to disparities, thus ensuring that the differences actually observed were primarily disease-specific. This allowed for clearly defined roles for such identified modifiable risk factors to include hypertension, smoking, diabetes mellitus, dyslipidemia, and lack of physical activity, all that were significantly more common with cases. The result only served to re-empower existing theories as for the central role of the involved modifiable lifestyle and health variables in CHF progression while simultaneously identifying the need for targeted preventive approaches. In practice, this study revealed the need to manage modifiable risk factors early and aggressively in patients with CHF in order to decrease their hospitalization burden and enhance the survival outcomes. Significant clinical differences, including hospitalization, mortality, and ejection fraction, have shown to be of serious health burdens caused by the disease of CHF, establishing the prognostic worth of ejection fraction as a protective marker. Such strong associations of hypertension, smoking, and diabetes mellitus with poor outcomes underscored the urgent need for multilevel intervention programs targeted to these modifiable risk factors. Findings regarding physical inactivity and unhealthy diet as contributing to the progression of CHF pointed out potential benefits from lifestyle modification programs.

The novelty of this study is that it assesses, for the first time, both risk factors and clinical outcomes in CHF, with a robust case-control analysis bridging gaps in understanding interplay between demographic, modifiable, and non-modifiable factors. Employing multivariate logistic regression and survival analysis, the study helped provide well-structured insights into prediction and protection factors which influence CHF outcomes. Unlike most previous studies that mainly focused on individual factors, this research introduced an integrated approach linking clinical and lifestyle factors with disease progression and outcomes. The emphasis placed on ejection fraction as a protective factor and the stringent identification of hospitalization patterns further added to what made the study different, paving the way for more targeted and evidence-based management strategies in CHF care.

As more persons get therapy, CHF prevalence grows even while other reports show that incidence rates have steadied. Neither an improvement in the quality of life nor a drop in the hospitalisation rates of CHF patients have corresponded with this increase. The Global Health Data Exchange registry estimates that there are currently 64.34 million instances of CHF worldwide, which translates to 9.91 million years lost to disability (YLDs) and $346.17 billion in medical expenses [12, 13].

One key element influencing the prevalence of CHF is age. Age-related increases in heart failure (HF) prevalence are reported for all classifications and causes. There was an increase from 8 per 1000 guys aged 50–59 to 66 per 1000 males aged 80–89, according to data from the Framingham Heart Study [14]. In men over 65, the incidence of heart failure doubles with each decade of life; in women, the incidence triples over the same age range. Men are more prevalent than women to suffer from heart disease and CHF worldwide [15, 16].

According to the global register, there is a racial disparity: Black individuals had a 25% larger prevalence of heart failure than White ones [2, 17, 18, 19]. HF continues to be the major cause of hospitalisation for the elderly and is responsible for 8.5% of cardiovascular-related deaths in the US [17].

Globally, there are analogous patterns in the epidemiology of HF, with incidence expanding significantly with advancing age, metabolic risk factors, and sedentary lifestyles. Ischemic cardiomyopathy and hypertension are the two leading causes of heart failure in poor nations [18]. According to a handful of studies, the incidence of HF has increased higher in younger individuals than in older people, which may be related to the expanding metabolic syndrome burden in younger populations [19, 20]. Additionally, data suggest that even among individuals with HFpEF, a large fraction of HF patients is young (less than 65 years old) [21, 22].

According to past studies, the elevated risk of cardiovascular disease due to diabetes and hypertension diminishes with age [23, 24]. According to the CALIBRE study, there is an age-related drop in the relative risk of 12 cardiovascular disorders linked to hypertension [25]. An earlier development of type 2 diabetes also raises the likelihood of death, as per several Swedish research [23, 26]. Previous study was hampered by its emphasis on macrovascular illness, dearth of documented heart failure diagnoses, and focus on one or a small number of risk variables [22, 23, 24, 25, 26].

A number of studies mentioned in literature can be assessed and drawn parallels from when analyzing our observations [27, 28, 29, 30, 31, 32, 33, 34, 35, 36]. Diabetes mellitus and hypertension were found to be substantial risk factors for CHF in both our study and Tromp *et al*.’s [27] investigation. Tromp *et al*. [27] recognised that these risk variables affected younger people more than older people, but our research did not account for age, so age-specific effects might have gone missed. The significance of social risk factors (SRFs) include education, social isolation, and area-deprivation index was underlined by Savitz *et al*. [28]. These aspects were not specifically looked at in our study. This means that social determinants might influence CHF outcomes more than our research demonstrated.

In keeping with the findings of Thomsen *et al*. [29], who identified an increase in hospitalisations among HF patients with hyperkalemia, our investigation demonstrated that CHF cases had a greater mean number of hospitalisations and longer hospital stays than controls. Both findings indicate how much CHF costs in terms of healthcare. Thomsen *et al*. [29] also highlighted the distinct impacts of hyperkalemia, which our study did not address. Both our study and that of Rigatto *et al*. [30] indicated that diabetes and ageing are major risk factors for CHF. Although our study did not directly address it, Rigatto *et al*.’s [30] focus on renal transplant patients indicated a larger prevalence of CHF in this cohort, suggesting a heightened risk.

The cardiovascular risks connected to particular vocations were explored by Fukai *et al*. [31], who identified greater risks in some employment categories. Occupational risk indicators were not taken into account in our study, suggesting a possible issue for future inquiry. Suboptimal medication adherence and proteinuria were reported to be substantial risk factors for CHF by Oguntade and Ajayi [32], with adherence being the most critical component. Another problem in our research is that, although our study focused on smoking and hypertension, it did not look at medication adherence or proteinuria.

The assessed findings demonstrated significant discrepancies between CHF patients and controls linking clinical outcomes and modifiable risk variables, demonstrating the major impact that hypertension, diabetes mellitus, dyslipidemia, smoking, and physical inactivity were playing in CHF development. Such findings are matched to other studies for example, Tromp *et al*. [27], which underlined the impact of such risk factors-particularly among younger persons. Despite the lack of age stratification in our study, studies done by Tromp *et al*. [27] underlined that therapies that target the management of CHF should also strive for age-grouped effects. Moreover, the relationships revealed between the risk factors indicated and higher hospitalization, ICU admissions and mortality concord well with those obtained by Thomsen *et al*. [29] in reporting significant health burden related to CHF. Moreover, reliance on self-reported data about the lifestyle behavior that is the focus of this study may indicate some memory bias that may have understated the true prevalence of these factors.

The patterns are mirrored to the rest of the world’s epidemiology in terms of CHF. This is through ageing age, metabolic risk factors, and sedentary lifestyles leading an increased illness burden [18, 19]. The Framingham Heart Study indicated an exponential increase in the prevalence of CHF with age, similarly consistent with our findings of increased hospitalizations and fatality rates in the older groups [14]. While CHF is historically considered a disease of the elderly, new evidence, such as in studies by Rigatto *et al*. [30] and analysis from HFpEF patients [21, 22], progressively pointed to increased loads in younger individuals with metabolic syndrome. This finding highlights the necessity of prevention including all age groups, considering the increased tendency toward obesity and diabetes in the younger population [19, 20]. Our study further reinforces this need by the identification of modifiable risk factors that can be intervened upon with public health interventions to retard the course of CHF and accompanying cost burden [12, 13].

Our study certainly has strengths in some areas, there are also some elements that need additional investigation. Emerging as key contributory factors in other studies, such as those of Savitz *et al*. [28], were social determinants of health such education, social isolation, and area deprivation indices. These were not looked at in the analysis and may well have meant that this broader context is ignored. Occupational risks of interest to Fukai *et al*. [31] were also not accounted for and are another essential facet in understanding CHF risk. Another element that was not addressed in the study was suboptimal adherence to medication, which is undoubtedly one of the variables that lead to the progression of CHF according to Oguntade and Ajayi [32]. This area so appears as a prospective future subject of inquiry. Moreover, our findings on admission rates are similar with Thomsen *et al*. [29], who in turn revealed an increased admission rate by this comorbidity, however our study could not analyse the matter on hyperkalemia, which may be another clue towards the management method of CHF.

Our findings accord with those of Savitz *et al*. [28], which pointed out that the progression of CHF is predominantly driven by hypertension. Their study, however, gave a more robust background than ours since it accounted in the temporal increase in obesity and smoking prevalence, which we did not specifically factor in our investigation. Earlier studies, such as by Oguntade and Ajayi [32], have also emphasised on the role of proteinuria and adherence to medication, which were not evaluated within the scope of this study. These are some significant weaknesses that future studies should address to have a better knowledge of the course of CHF. Furthermore, while our analysis validates the significance of established risk factors in CD, the results of Tromp *et al*. [27] and Rigatto *et al*. [30] underline the need of looking at age-stratified and context-specific changes in risk factor prevalence and outcomes.

The results of our CHF study were both similar and different to the studies by Fitchett *et al*. [33], Lawson *et al*. [34], He *et al*. [35], and Djoussé *et al*. [36]. Our study and Fitchett *et al*. [33] highlighted the importance of cardiovascular risk factors like hypertension, diabetes mellitus, and smoking in predicting adverse outcomes. Although Fitchett *et al*. [33] highlighted consistent reductions in cardiovascular events with empagliflozin across various levels of baseline cardiovascular risk, our study was on identifying those risk factors rather than looking at pharmacological interventions.

Lawson* et al*. [34] showed sociodemographic trends where differences in age, socioeconomic status, and ethnic disparities are partially replicated here. While no socioeconomic status differences were observed in our cohort, the impact of the non-modifiable factor, age, was consistent with Lawson *et al*. [34] in that they found significant associations in these populations. Unlike their study, however, ours did not stratify findings by ethnicity or examine longitudinal trends.

The prognostic significance of ejection fraction identified in our study is consistent with He *et al*.’s [35] work on race and socioeconomic factor stratification of cardiovascular risk over two decades. Both He *et al*.’s [35] and our study highlights critical risk factors such as BMI and systolic blood pressure, although He *et al*. [35] provided a much more detailed temporal analysis while our study offered cross-sectional data. Similarly, both our findings and the study from Djoussé *et al*. [36] emphasized hospitalization burden of CHF. However, Djoussé *et al*. [36] targeted the effects of vitamin D and n-3 fatty acid supplementation, with no significant impact on initial hospitalizations but potential benefits for recurrent hospitalizations with n-3 supplementation. In contrast, our study concentrated on clinical and demographic predictors without investigating nutritional or supplemental interventions.

### 4.1 Limitations

There were different limits on this inquiry. First, it was hard to prove a relationship between the risk factors that were observed and CHF because of the cross-sectional design. Second, recall bias may have been established by using self-reported data for part of the data collection, particularly when it comes to lifestyle traits like smoking and physical inactivity. Third, the study population was picked from a single geographic region, which would have limited how broadly the results might be applied. Fourth, information about past medical conditions and medication use may have been erroneous or incomplete as a result of the secondary data’s reliance on medical records. Furthermore, even after being controlled for in the multivariate analysis, confounding variables might still have had an impact on the observed associations. Lastly, the study did not properly explore the likely influence of psychosocial and socioeconomic factors, which may be crucial in the aetiology and therapy of CHF. Patient compliance with treatment regimens and lifestyle modifications was not assessed, potentially influencing outcomes. Variations in treatment protocols, such as the use of medications, were not accounted for, which could confound the results. Although demographic variables were balanced, unmeasured factors like socioeconomic status, healthcare access, and comorbidities may have introduced bias. The reliance on BMI as a measure of body composition and hospital records for outcomes may have limited the depth and accuracy of the analysis. Additionally, the cross-sectional design prevented causal inferences, highlighting the need for longitudinal studies to validate these associations.

### 4.2 Recommendations Pertaining to Practice and Future Implications

A number of recommendations might be made in light of the data to enhance the prognosis of CHF patients. First and foremost, smoking cessation, diabetes mellitus, hypertension, and dyslipidemia should all be addressed with specific therapy. It is vital to adjust lifestyle, stick to medicine regimen, and undertake frequent monitoring. In addition to boosting exercise, proper dietary practices can help lower risk factors. Healthcare systems should strengthen their patient education emphasising the significance of regular medication compliance and routine follow-ups. One important approach for early intervention identification of high-risk patients is screening for a family history of cardiovascular disease.

Furthermore, structured discharge planning and increased outpatient management are crucial to reduce hospitalisation rates and length of stay. Integrating multidisciplinary care teams will enable them to offer total management and assistance. Personalised care regimens should also be devised for high-risk individuals, with an emphasis on diabetes and smoking cessation in addition to ejection fraction monitoring. Patients with CHF can considerably boost their quality of life and clinical results by putting these guidelines into practice.

## 5. Conclusion

We discovered that, in comparison to controls, patients with CHF had a much increased prevalence of modifiable risk factors, such as smoking, diabetes mellitus, dyslipidemia, hypertension, and physical inactivity. Adverse clinical outcomes, including greater rates of hospitalisation, longer hospital stays, higher rates of rehospitalization within 30 days, and more frequent ICU admissions, were strongly linked with these risk variables. Patients with CHF also showed greater cardiovascular and all-cause mortality, higher MACE incidence, worse quality of life, and inferior functional status. These results underscore the urgent need for tailored treatments to regulate and diminish these risk factors, increase patient outcomes, and lessen the cost burden of CHF-related healthcare.

## Availability of Data and Materials

All the data generated during the study is presented in the results section.
